# A Comprehensive Functional Analysis of *NTRK1* Missense Mutations Causing Hereditary Sensory and Autonomic Neuropathy Type IV (HSAN IV)

**DOI:** 10.1002/humu.23123

**Published:** 2016-11-26

**Authors:** Samiha S. Shaikh, Ya‐Chun Chen, Sally‐Anne Halsall, Michael S. Nahorski, Kiyoyuki Omoto, Gareth T. Young, Anne Phelan, Christopher Geoffrey Woods

**Affiliations:** ^1^ Cambridge Institute for Medical ResearchUniversity of CambridgeCambridgeCB2 0XYUK; ^2^ Molecular Genetics LaboratoryAddenbrooke's HospitalCambridgeUK; ^3^ Neuroscience and Pain Research UnitPfizer LtdGreat AbingtonUK; ^4^ Stratified MedicalLondonNW1 1LWUK

**Keywords:** CIPA, TRKA, NGF, pain, neuropathy

## Abstract

Hereditary sensory and autonomic neuropathy type IV (HSAN IV) is an autosomal recessive disorder characterized by a complete lack of pain perception and anhidrosis. Here, we studied a cohort of seven patients with HSAN IV and describe a comprehensive functional analysis of seven novel *NTRK1* missense mutations, c.1550G >A, c.1565G >A, c.1970T >C, c.2096T >C, c.2254T >A, c.2288G >C, and c.2311C >T, corresponding to p.G517E, p.G522E, p.L657P, p.I699T, p.C752S, p.C763S, and p.R771C, all of which were predicted pathogenic by in silico analysis. The results allowed us to assess the pathogenicity of each mutation and to gain novel insights into tropomyosin receptor kinase A (TRKA) downstream signaling. Each mutation was systematically analyzed for TRKA glycosylation states, intracellular and cell membrane expression patterns, nerve growth factor stimulated TRKA autophosphorylation, TRKA‐Y496 phosphorylation, PLCγ activity, and neurite outgrowth. We showed a diverse range of functional effects: one mutation appeared fully functional, another had partial activity in all assays, one mutation affected only the PLCγ pathway and four mutations were proved null in all assays. Thus, we conclude that complete abolition of TRKA kinase activity is not the only pathogenic mechanism underlying HSAN IV. By corollary, the assessment of the clinical pathogenicity of HSAN IV mutations is more complex than initially predicted and requires a multifaceted approach.

## Introduction

Pain is a physiological protective mechanism that is essential for the detection and prevention of contact with noxious stimuli present in all multicellular organisms [Dubin and Patapoutian, 2010]. Such noxious stimuli are detected by specialized sensory neurons called nociceptors. During development, specification toward a nociceptive phenotype relies on the expression of the receptor called tropomyosin receptor kinase A (TRKA) for the final transition from mitotic precursor to post‐mitotic nociceptor [Marmigere and Ernfors, 2007]. Further underlining importance of TRKA in human pain is that either its deficiency or deficiency of its ligand, nerve growth factor (NGF), results in an inherited form of congenital painlessness named Hereditary Sensory and Autonomic Neuropathy type IV (HSAN IV) and HSAN V, respectively. In addition, novel antagonists or therapeutic antibodies against NGF‐TRKA signaling are showing considerable promise as analgesics in clinical trials [Bannwarth and Kostine, 2014; Spierings *et al*. 2014].

**Figure 1:**
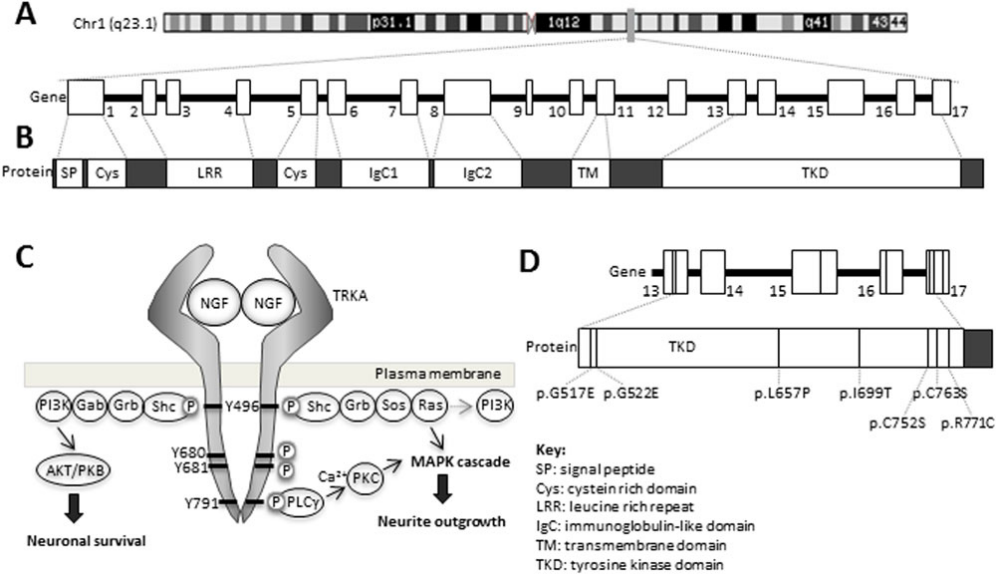
TRKA structure, signaling, and identification of novel *NTRK1* mutations in patients with HSAN IV. **A**: *NTRK1* located on chromosome 1q21‐q22, shown by the gray bar on the ideogram, consists of 17 exons and spans region of 25 kb. Inclusion of all 17 exons encodes for the neuronal specific isoform of TRKA. **B**: The extracellular domain of TRKA consists of a number of structural domains: a leucine rich repeat, flanked on either side by a cysteine rich domain, and two immunoglobulin‐like domains. The majority of the intracellular domain comprises the kinase domain. **C**: Signaling through TRKA is initiated through NGF binding, TRKA dimerization, and autophosphorylation of Y676, Y680, and Y681. Subsequent phosphorylation of Y496 and Y791 leads to activation of downstream signaling cascades which drive neuronal differentiation and survival. **D**: Seven individuals were ascertained with a standard HSAN IV phenotype. The identified mutations were p.G517E, p.G522E, p.L657P, p.I699T, p.C752S, p.C763S, and p.R771C. Only the exons that code for the kinase domain are shown. TKD, tyrosine kinase domain.

The human TRKA protein is encoded by the gene *NTRK1* (MIM# 191315) located on chromosome 1q21‐q22 (Fig. 1A). The first isoform of *NTRK1* includes all exons and yields a 796 residue protein that is predominantly expressed in neuronal tissues and provides responsiveness to both NGF and neurotrophin 3 [Barker *et al*. 1993]. Exclusion of exon 9 in the second isoform results in a 790 residue protein that is primarily expressed in non‐neuronal tissues and binds NGF alone [Barker *et al*. 1993]. Previous studies have shown TRKA to be synthesized as an 87 kDa protein, which is immediately glycosylated to a 110 kDa form. Further glycosylation events occur to produce the mature and functional 140 kDa glycoprotein [Martin‐Zanca *et al*. 1989], which is then trafficked to the plasma membrane [Watson *et al*. 1999].

The TRKA receptor contains a single transmembrane domain that lies between the extracellular and intracellular domains. The extracellular domain consists of a number of structural motifs (Fig. 1B) which specifies binding to NGF, whereas the majority of the intracellular domain comprises a tyrosine kinase domain [Huang and Reichardt, 2003]. Intracellular signaling by TRKA is initiated by engagement with an extracellular NGF dimer, which causes the dimerization of two TRKA molecules (Fig. 1C) and autophosphorylation of the TRKA kinase domain tyrosine residuesY676, Y680, and Y681 [Cunningham *et al*. 1997]. The subsequent structural change allows binding to ATP and results in phosphorylation of Y496 and Y791 [Cunningham and Greene, 1998; Artim *et al*. 2012], which then act as adaptor sites for the downstream signaling molecules Shc and PLCγ, respectively, summarized in Figure 1C.

Recruitment of Shc [Dikic *et al*. 1995] and associated adaptor proteins to phosphorylated Y496 activates PI3K to promote neuronal survival [Datta *et al*. 1999; Yuang and Yanker, 2000]. Binding of other associated adaptor proteins to Shc activates the Ras/MAPK pathway [Dikic *et al*. 1995; MacDonald *et al*. 2000] (Fig. 1C). PLCγ bound to phosphorylated Y791 results in an increase in Ca^2+^ levels and activation of Ca^2+^/calmodulin‐regulated protein kinases [Obermeier *et al*. 1993]. The Ras/MAPK and PLCγ pathways converge to lead to transcription of genes that specifically drive neuronal differentiation [Riccio *et al*. 1999; Lonze *et al*. 2002].

HSAN IV (OMIM #256800) is a rare autosomal recessive disorder that is caused by a failure of nociceptive and sympathetic neuron development. One of the characteristic features of HSAN IV is a congenital and complete insensitivity to both superficial and deep painful stimuli. Patients are prone to oral injuries as well as multiple accidental injuries such as burns, falls, and bone and joint fractures [Amano *et al*. 1998; Bar‐On *et al*. 2002]. Further key features of HSAN IV are a deficit of temperature sensing [Ismail *et al*. 1998; Axelrod 2002] and developmental delay which becomes apparent within the first decade [Rosemberg *et al*. 1994]. Recently it has been shown that lack of TRKA signaling increases the susceptibility of patients to infections, in particular to *Staphylococcus aureus*, through a monocyte/macrophage‐specific NGF/TRKA pathway [Beiglman *et al*. 2009; Hepburn *et al*. 2014].

Many different mutations have been identified in HSAN IV patients, mostly nonsense mutations, but also missense, small insertions, small deletions, and splicing variants. Only a small number of missense mutations have previously been functionally analyzed (review of information in Supp. Table S1), the mutations p.G516R, p.G571R, p.R643W, p.R648C, p.G708S, and p.R774P (numbering based on RefSeq ID NP_001012331). All showed diminished kinase activity and absent autophosphorylation [Greco *et al*. 1999; Greco *et al*. 2000; Mardy *et al*. 2001; Miranda *et al*. 2002], and in addition, the extracellular domain mutations p.L93P and p.L213P were aberrantly processed and showed reduced kinase activity [Mardy *et al*. 2001; Miranda *et al*. 2002].

We have identified seven novel missense mutations in *NTRK1* from patients with HSAN IV. Extensive functional analysis of each mutation revealed a diverse and novel range of results and suggests multiple pathogenic mechanisms underlie HSAN IV.

## Methods

### Molecular Genetics, Sequencing, and Segregation Studies

The seven missense mutations described in this study were sequentially ascertained through an NHS genetics service. Patients were accepted for analysis if they had the minimal clinical characteristics of both a congenital pain insensitivity and cognitive delay. Sequencing was by standard Sanger analysis of each exon of *NTRK1* (RefSeq ID NM_002529.3) using a customized set of primers designed to work under identical PCR conditions (primer sequences are available on request). The *NGF* gene (RefSeq ID NM_002506.2) was also sequenced using a set of primers designed to work under identical conditions.

The conservation of the mutated residues was analyzed using the multiple sequence alignment tool Clustl Omega version 1.2.2 (McWilliam *et al*. 2013) (http://www.ebi.ac.uk/Tools/msa/clustalo/). Pathogenicity of mutations was predicted by PolyPhen‐2 [Adzhubei *et al*. 2010] (http://genetics.bwh.harvard.edu/pph2/) and by SIFT [Kumar *et al*. 2009] (http://sift.jcvi.org/www/SIFT_enst_submit.html). For all tools, default settings were used. Our chromosome and mutation lab results have been uploaded on DECIPHER (https://decipher.sanger.ac.uk), where they are freely available, under an agreement with NHS‐CAM and also on LOVD database (http://grenada.lumc.nl/LSDB_list/lsdbs/NTRK1).

### Cloning of Expression Constructs

The construct for wild‐type full‐length TRKA was obtained from IMAGE clones (Source BioSciences, Cambridge, UK). A C‐terminal GFP tagged TRKA construct was generated by introducing full‐length *NTRK1* cDNA into the pEGFP‐N1 cloning vector (Clontech, Mountain View, CA, USA) as described previously [Mitchell *et al*. 2012], and henceforth referred to as wild‐type TRKA‐GFP. GFP tagged TRKA had a similar activity to untagged TRKA (Supp. Fig. S1). All the point mutations were introduced using the QuikChange II Site‐directed Mutagenesis Kit (Agilent Technologies, Santa Clara, CA, USA).

### Cell Culture and Conditions

HEK‐293, HeLa, and SH‐SY5Y cells were cultured in complete DMEM supplemented with 10% FBS, 2 mM l‐glutamine and 100 μg/ml penicillin and 100 μg/ml streptomycin, at 37°C and 5% CO_2_.

For localization studies, HEK‐293 cells were plated on poly‐l‐lysine coated coverslips at a seeding concentration of 1 × 10^5^ cells/ml. For neurite outgrowth, SH‐SY5Y cells were plated on poly‐l‐lysine coated coverslips at 5 × 10^4^ cells/ml. Cells were transiently transfected with 500 ng DNA with Fugene HD transfection reagent (Promega, Madison, WI, USA).

For glycosylation assessment, HEK‐293 cells were plated at a seeding concentration 2.5 × 10^5^ cells/ml. For phospho‐ELISA assays and calcium imaging, HeLa cells were plated at 1.25 × 10^5^ cells/ml. For calcium imaging, cells were plated on poly‐l‐lysine coated 35 mm glass bottom dishes. In all cases, cells were transiently transfected with 2.5 μg DNA using Fugene HD for 24 hr.

### Colocalization Analysis

Twenty‐four hours post‐transfection, HEK‐293 cells were washed in PBS and fixed in ice‐cold methanol. Cells were stained with GFP antibody (Abcam, Cambridge, UK; ab6556, 1:1000) and Na^+^/K^+^ ATPase α1 subunit antibody (Abcam; ab7671, 1:300). Alexa‐488 and Alexa‐546 conjugated secondary antibodies were purchased from Invitrogen, Carlsbad, CA, USA (1:1,000). All images were acquired with an LSM710 laser‐scanning META confocal microscope (Carl Zeiss, Oberkochen, Germany) using a ×63 oil‐immersion objective.

Image analysis was performed using the Volocity image analysis software (PerkinElmer, Waltham, MA, USA). Colocalization was quantified in terms of Manders M1 coefficient. The proportion of GFP at the membrane was quantified by measuring the GFP at the plasma membrane and dividing by the total GFP fluorescence.

### Glycosylation Assessment

Transfected HEK‐293 cells were washed with 1× PBS and scraped in RIPA buffer (Tris pH 7.4, NaCl 150 mM, EDTA, 0.5 mM, 1% Triton), containing protease inhibitors (Roche Applied Sciences, Penzberg, Germany). Lysates were cleared by centrifugation at 16,000*g* at 4°C for 25 min and levels of total cellular protein tested using the DC protein assay kit (BioRad, Hercules, CA, USA). Total protein (25 μg) was treated with PNGase (NEB) following manufacturer's protocol or was left untreated.

PNGase‐treated and ‐untreated protein lysate were separated using 6% Tris‐glycine gels (Invitrogen) and transferred to PVDF membrane (Millipore, Billerica, MA, USA). Blots were then probed with GFP (D5.1) XP Rabbit mAb #2956 (Cell Signaling Technologies, Danvers, MA, USA; 1:1,000) and lamin antibody (Abcam; ab133741, 1:1000). Secondary antibodies were purchased from Dako (Santa Clara, CA, USA) and signal was detected using the chemiluminescent HRP substrate (Millipore). Densitometry was performed with ImageJ software (NIH).

### Phospho‐TRKA Assays

Twenty‐four hours post transfection, HeLa cells were serum starved in DMEM supplemented with 1% FBS at 37°C for 1 hr. Cells were then treated with 100 ng/ml NGF (CST) for 5 min at 37°C or were left untreated and washed in 1× Tris buffered saline. Lysate was collected as mentioned previously, with one alteration: RIPA buffer was supplemented with a 1:100 dilution of phosphatase inhibitor cocktails 2 and 3 (Sigma, St Louis, MO, USA).

The autophosphorylation and Y496 phosphorylation levels were determined using the PathScan® Phospho‐TRKA Sandwich ELISA 674/675 Kit (CST) and PathScan® Phospho‐TRKA Tyr490 Sandwich ELISA kit (CST), respectively, following manufacturer's protocol. Total TRKA levels in the lysates were also measured using PathScan® Total TRKA Sandwich ELISA kit (CST).

The relative levels of phosphorylated TRKA were calculated by dividing phosphorylated values by total TRKA values. This was then normalized to the relative phosphorylation of wild‐type transfected cells without NGF treatment.

### Calcium Imaging

Twenty‐four hours post‐transfection, calcium imaging was performed on HeLa cells using Rhod‐3‐AM calcium imaging kit (Invitrogen) following manufacturer's protocol. Images were acquired with an LSM710 laser scanning META confocal microscope (Carl Zeiss) using a ×20 objective and maximum pinhole aperture of 600 μm. Two line averages were performed for each frame with images taken every 4 sec.

At *t* = 48 sec, 20 μl of HBSS media was added, at *t* = 140 sec, 20 μl NGF (final concentration of 100 ng/ml) was added to the cells. The response to NGF was monitored for 10 min, after which at *t* = 820 sec, 100 ng/ml ionomycin was added. For PLCγ inhibition studies, WT transfected cells were treated with 3 μg/ml of U73122 (Calbiochem, Billerica, MA, USA) for 30 min prior to NGF stimulation.

Image analysis was performed with the Volocity 3D Image Analysis Software using green cells that did not spontaneously fluoresce and had an ionomycin response.

### Neurite Outgrowth Assays

Twenty‐four hours post transfection, SH‐SY5Y cells were treated with 100 ng/ml NGF in DMEM supplemented with 5% FBS for 9 days, with media being replaced every 3 days. Cells were then washed in PBS and fixed in 4% paraformaldehyde and were imaged with an LSM510 laser scanning META confocal microscope (Carl Zeiss) using a ×40 immersion oil objective. Cells were analyzed using the ImageJ software. A neurite was identified as being more than twice the cell body length, where the longest dimension was used [Nahorski *et al*. 2015]. Total neurite length per cell was also quantified where all extensions were measured and divided by the total number of cells counted [Nahorski *et al*. 2015].

### Structural Modeling

The homology model of the human TRKA tyrosine kinase domain (UniProtKB: P04629) was created using in‐house Pfizer software and Modeler 9v8 (http://salilab.org/modeller/) [Sali and Blundell, 1993] using human TRKA structures 4AOJ_A, 4GT5, 4PM, 4PP, 4PS, and 4PT, deposited in the Protein Data Bank as templates, http://www.rcsb.org/pdb/home/home.do, as previously described [Veale *et al*. 2014].

### Statistical Analysis

The data show the mean ± standard error of the mean (SEM). Statistical significance was calculated using a one‐way ANOVA, followed by unpaired, two sided Student's *t*‐test using a Bonferroni corrected adjusted *P* value. Significance was set at ^*^ *P* < 0.05, ^**^ *P* < 0.01, or ^***^ *P* < 0.001.

## Results

### Putative Mutations in *NTRK1*

Seven cases with a typical HSAN IV phenotype, in which parents had no features of HSAN IV, were referred to the East Anglian NHS Genetics Services for HSAN IV analysis and were sequentially identified. The probands ages were from 3 to 15 years. They had no evidence of having pain or temperature perception, had cognitive global delay ranging from mild to moderate. They did not sweat and usually had ichthyotic areas of skin with troublesome pruritus. Those over 10 years of age had evidence of Charcot's joints in the lower limbs, and had had at least one significant episode of *S. aureus* infection. (For full clinical and mutation details, see Supp. Table S2.) Four cases were consanguineous, whilst three cases were from singleton families. Through sequencing analysis, we found three individuals who were homozygous for different *NTRK1* mutations, two cases with a heterozygous missense mutation and a heterozygous nonsense mutation and one case with two heterozygous missense mutations. Sequencing of both parents in all cases confirmed that the mutations segregated as expected for an autosomal recessive disorder. *NGF* was also sequenced and contained no mutations. The novel identified mutations were c.1550G>A, c.1565G>A, c.1970T>C, c.2096T>C, c.2254T>A, c.2288G>C, c.2311C>T (RefSeq ID NM_002529.3), corresponding to the TRKA protein changes p.G517E, p.G522E, p.L657P, p.I699T, p.C752S, p.C763S, and p.R771C, respectively (Fig. 1D and Supp. Fig. S2). Nucleotide numbering uses +1 as the A of the ATG translation initiation codon in the reference sequence, with the initiation codon as codon 1. There are no variations at these positions recorded in the 1000 Genomes Server, dbSNP (http://www.ncbi.nlm.nih.gov/SNP/), the Exome Variant Server (http://evs.gs.washington.edu/EVS/), or the ExAC database (http://exac.broadinstitute.org). p.G522R (called p.G516R in the article) has previously been reported in a HSAN IV case [Mardy *et al*. 2001]. Interestingly, all of the mutations identified here were located intracellularly within the kinase domain (Supp. Figs. S2 and S3), as is the case for most of the previously described missense mutations in *NTRK1*. All mutations were in evolutionarily conserved residues and were predicted to be pathogenic by Polyphen and SIFT (Supp. Fig. S3). As none of the mutations would be expected to cause a loss of or instability of the TRKA protein, a number of different studies were undertaken to determine the pathogenic mechanism. Table 1 summarizes the results.

**Table 1:** Summary of Functional Characterization of New TRKA Mutations

***NTRK1* mutation**	**TRKA protein**	**Full glycosylation**	**Membrane expression**	**Autophos‐phorylation**	**Y496 phos‐phorylation**	**PLCγ activation**	**Neurite outgrowth**
c.1550G > A	p.G517E	No effect	No effect	No effect	No effect	Abolished	Reduced
c.1565G > A	p.G522E	No effect	No effect	Abolished	Abolished	Abolished	^‐^
c.1970T > C	p.L657P	Reduced	Reduced	Abolished	Abolished	Abolished	Abolished
c.2096T > C	p.I699T	Reduced	Reduced	Abolished	Abolished	Abolished	^‐^
c.2254T > A	p.C752S	No effect	No effect	No effect	No effect	No effect	No effect
c.2288G > C	p.C763S	Reduced	Reduced	Reduced	Reduced	Reduced	Reduced
c.2311C>T	p.R771C	Reduced	Reduced	Abolished	Abolished	Abolished	^‐^

GFP tagged constructs of wild‐type TRKA and each mutation were used to asses pathogenicity (RefSeq ID NM_002529.3, NP_002520).

### Glycosylation Changes in TRKA Mutants

**Figure 2:**
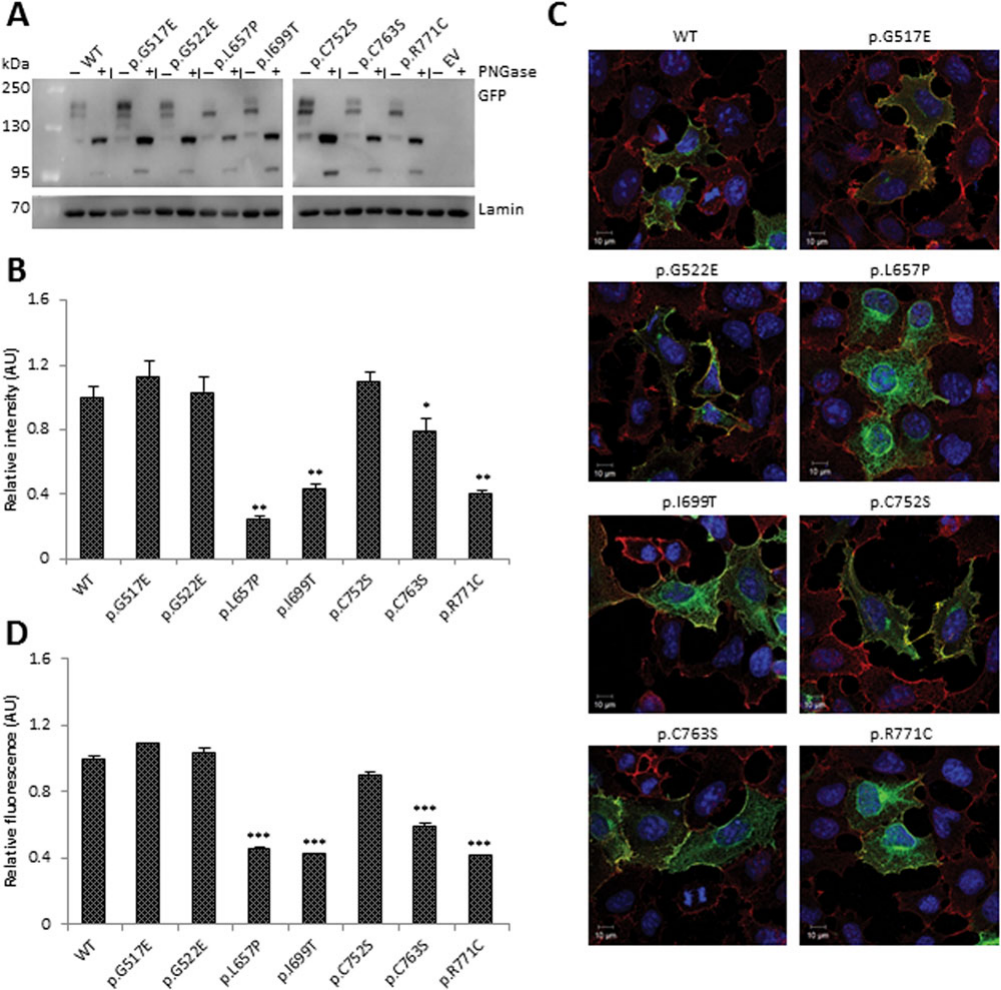
Expression, processing, and localization of wild‐type and mutant TRKA. **A**: Expression of C‐terminal GFP tagged wild‐type and mutant TRKA constructs in transfected HEK‐293 cells. The lanes “+” and “−” represent lysates treated with/without PNGase respectively. Lamin B1 was included as a loading control. Representative image of *n* = 3 is shown. WT, wild‐type; EV, empty vector. **B**: Quantification of relative levels of fully glycosylated 170 kDa TRKA‐GFP protein to the levels of partially glycosylated 130 kDa TRKA‐GFP protein. **C**: Localization of TRKA‐GFP wild‐type and mutant proteins in transfected HEK‐293 cells. The cells were stained against GFP (green) and the plasma membrane marker Na^+^/K^+^ ATPase (red). Wild‐type TRKA and all the mutants show membrane expression. **D**: Relative levels of GFP at the membrane compared with the levels of GFP in the cytoplasm. Graph represents mean values of *n* = 3, with at least 15 cells in each repeat, and error bars represent standard error of the mean. Statistical differences are indicated as ^*^ *P* < 0.05, ^**^ *P* < 0.01 (one‐way ANOVA, followed by Student's *t*‐test using a Bonferroni adjusted *P* value).

We first determined whether the mutations affected protein synthesis or post‐translational modification. C‐terminal tagged GFP wild‐type and mutant constructs were transfected into HEK‐293 cells and lysates were collected and were either treated with peptide‐N‐glycosidase (PNGase) or were left untreated. PNGase treatment of wild‐type and mutant lysate removed N‐glycosylation to yield an unglycosylated backbone of 117 kDa (Fig. 2A). Untreated lysate of wild‐type TRKA‐GFP had two protein bands: the fully glycosylated 170 kDa product and the partially glycosylated 140 kDa product. All of the mutants clearly showed the 170 kDa fully glycosylated and 140 kDa partially glycosylated products. However, compared with wild‐type TRKA‐GFP, p.L657P showed a fourfold reduction in the fully glycosylated form, whilst p.I699T and p.R771C showed a 2.5‐fold reduction (^**^ *P* < 0.01; Fig. 2B). p.C763S also showed a 1.3‐fold reduction in the 170 kDa form (^*^ *P* < 0.05; Fig. 2B). p.G517E, p.G522E, and p.C752S showed a glycosylation pattern that was similar to wild‐type TRKA‐GFP.

### Localization of TRKA‐GFP Mutant Proteins

We then determined the localization of the mutant TRKA‐GFP proteins to see whether the glycosylation changes observed affected the subcellular localization. HEK‐293 cells were transfected with wild‐type and mutant constructs and were stained with antibodies against GFP, shown in green, and the plasma membrane marker Na^+^/K^+^ ATPase α1 subunit, shown in red (Fig. 2C). As previously observed, wild‐type TRKA‐GFP was present around the periphery of the cells and colocalized with Na^+^/K^+^ ATPase staining (Supp. Fig. S4). Staining of wild‐type TRKA‐GFP demonstrated that 45% of the protein was located at the cell surface with the remainder being located intracellularly (Fig. 2D). This was not significantly different in cells expressing the p.G517E, p.G522E, or p.C752S mutations. Although the remaining mutant proteins colocalized at the plasma membrane, the proportion of GFP at the membrane was significantly reduced to approximately 20% to that of wild‐type TRKA‐GFP (Fig. 2D).

Although many of the mutations altered the glycosylation status of TRKA‐GFP and its localization, they did not completely block trafficking of the receptors to the plasma membrane. Therefore, we next questioned whether the mutants could activate various downstream signaling pathways.

### Autophosphorylation of TRKA‐GFP Mutant Proteins

**Figure 3:**
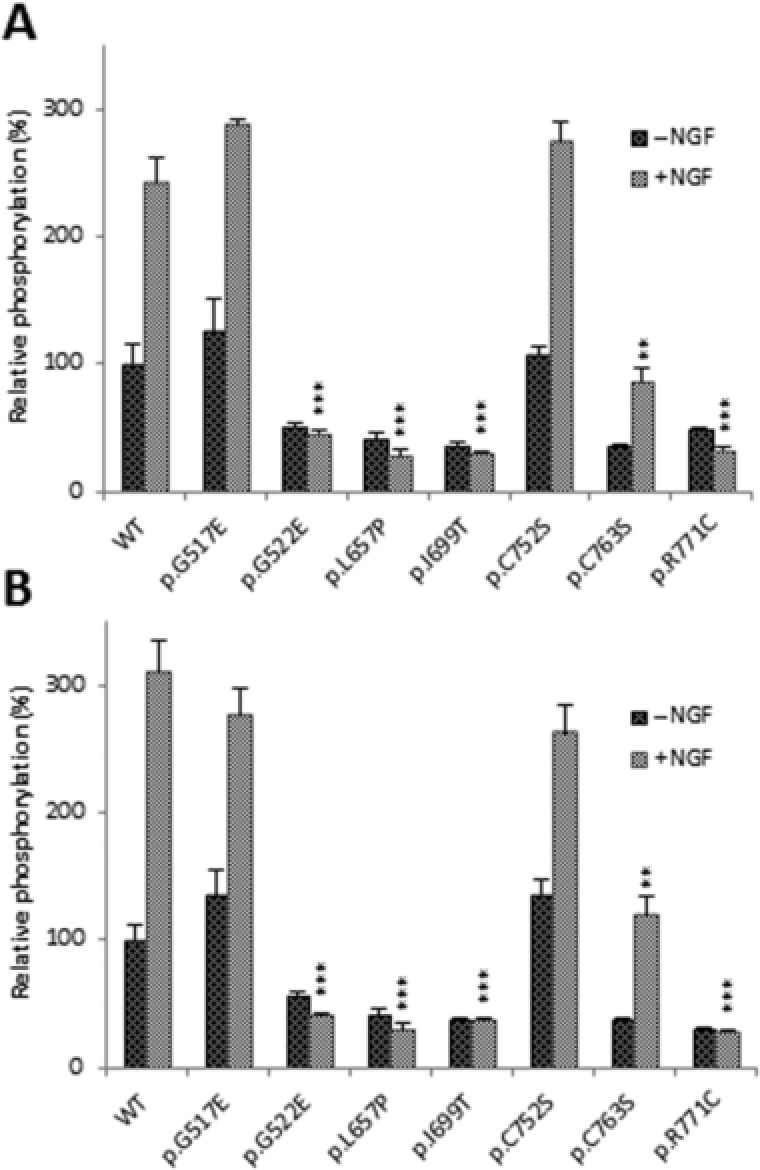
Relative Y680/681 and Y496 phosphorylation of mutant TRKA. **A**: Basal level of wild‐type and mutant TRKA‐GFP autophosphorylation in transfected untreated HeLa cells. Response to wild‐type and mutant TRKA‐GFP protein to 15 min stimulation with 100 ng/ml NGF. **B**: Basal level of Y496 phosphorylation in wild‐type and mutant TRKA proteins in untreated cells and after NGF stimulation. The bar graphs in A and B represents the mean values of *n* = 3 and error bars represents standard error of the mean. Statistical differences between induced phosphorylation of wild‐type and mutant TRKA are indicated as ^**^ *P* < 0.01 or ^***^ *P* < 0.001 (one‐way ANOVA, followed by Student's *t*‐test using a Bonferroni adjusted *P* value). WT, wild‐type; EV, empty vector.

We next looked at the effects of the mutations on autophosphorylation of TRKA‐GFP. HeLa cells were transfected with wild‐type and mutant constructs. The basal level of autophosphorylation of wild‐type TRKA‐GFP increased 2.5‐fold after NGF stimulation (Fig. 3A). There was a threefold reduction in basal levels of autophosphorylation in four of the mutants, namely p.G522E, p.L657P, p.I699T, and p.R771C (^***^ *P* < 0.001) and there was no induction of NGF‐stimulated TRKA‐GFP autophosphorylation (^***^ *P* < 0.001; Fig. 3A). There were no observable differences in the basal levels of autophosphorylation, nor induction of autophosphorylation after NGF stimulation in p.G517E and p.C752S. In p.C763S, there was a threefold reduction in basal levels of autophosphorylation and NGF stimulated autophosphorylation (^**^ *P* < 0.01).

### Y496 Phosphorylation of Mutant TRKA‐GFP Proteins

We then looked at the effect of the mutations on Y496 phosphorylation which is important for the recruitment of Shc and downstream signaling through PI3K and the Ras/MAPK pathway. HeLa cells were transfected with wild‐type and mutant constructs, and then NGF stimulated. The basal level of wild‐type TRKA‐GFP Y496 phosphorylation increased threefold upon NGF stimulation (Fig. 3B). The four mutants, p.G522E, p.L657P, p.I699T, and p.R771C, which showed defective autophosphorylation, did not induce Y496 phosphorylation after NGF stimulation (^***^ *P* < 0.001), whereas p.G517E and p.C752S did not alter basal nor NGF‐stimulated Y496 phosphorylation. In p.C763S, there was a threefold reduction in basal Y496 phosphorylation and a 2.5‐fold reduction in NGF‐stimulated Y496 phosphorylation (^**^ *P* < 0.01).

### Induction of PLCγ Pathway

**Figure 4:**
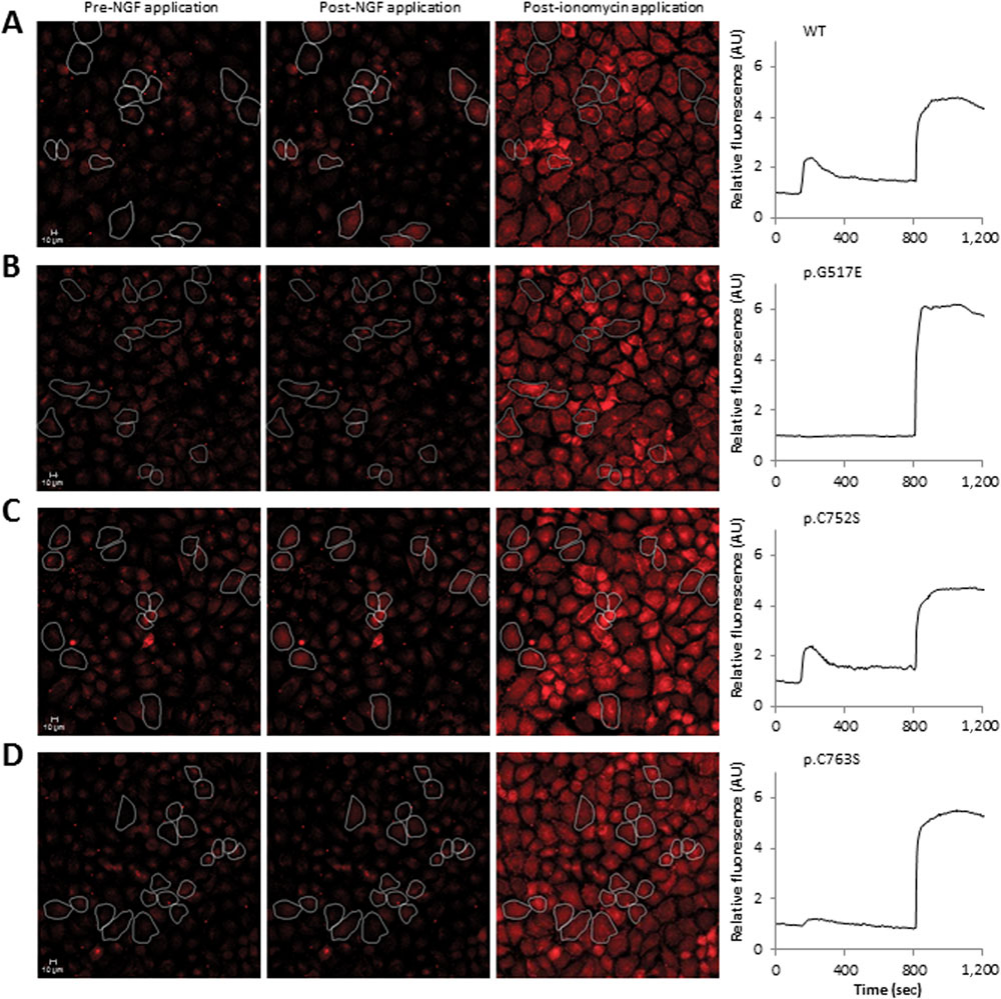
Effect of mutations on induction of PLCγ pathway. **A**: HeLa cells were transfected with wild‐type and mutant TRKA constructs and loaded with rhodamine‐3 and response to NGF was monitored. Ionomycin was used as the positive control. Only cells that did not spontaneously fluoresce and had an ionomycin response were used for analysis. WT, wild‐type. **B**: No significant increase in calcium levels was observed after NGF stimulation in p.G517E transfected cells. **C**: There was an increase in fluorescence in p.C752S transfected cells which peaked at *t* = 208. **D**: NGF stimulation on cells transfected with p.C763S caused an increase in fluorescence but the maximum value was reduced.

As p.G517E and p.C752S did not seem to have any effect upon trafficking or autophosphorylation, as described above, we determined their ability to activate the PLCγ pathway through monitoring calcium flux after NGF stimulation. We also tested the other mutants. HeLa cells were transfected and loaded with the calcium indicator dye rhodamine‐3. In wild‐type transfected cells, after NGF stimulation, there was an increase in basal fluorescence (Fig. 4A) to a maximum fluorescence of 2.5 AU at *t* = 208 sec (Supp. Fig. S5A). Furthermore, 90% of transfected cells responded to NGF (Supp. Fig. S5B). Treatment with U73122, a PLCγ inhibitor, abolished induction of NGF increase of calcium (Supp. Fig. S5C).

Fluorescence remained at basal levels in cells transfected with the mutant proteins that did not autophosphorylate or phosphorylate Y496 (Supp. Fig. S5A). NGF stimulation of p.G517E transfected cells did not induce an increase in red fluorescence (Fig. 4B), which remained at basal levels (^**^ *P* < 0.01) and the percentage of cells responsive to NGF was 3% (Supp. Fig. S5B). There was no observable difference in the calcium response in p.C752S transfected cells (Fig. 4C) compared with the response observed in wild‐type TRKA‐GFP proteins. In p.C763S transfected cells, basal fluorescence increased to a maximum value of 1.2 AU (Fig. 4D; ^**^ *P* < 0.01) and only 50% of cells were NGF responsive.

### Neurite Outgrowth

**Figure 5:**
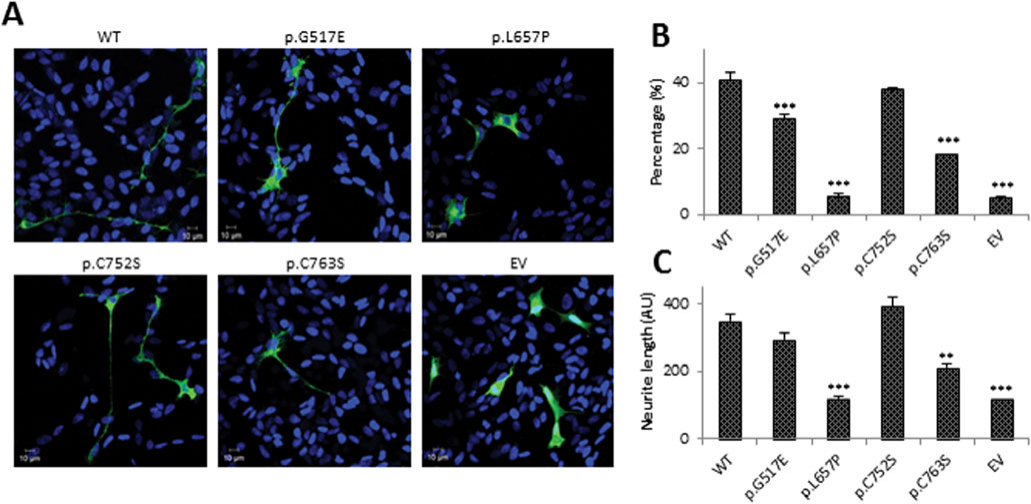
Differentiation ability of selected mutants. **A**: Representative images of transfected SH‐SY5Y cells treated with NGF for 9 days. WT, wild‐type; EV, empty vector. Green signal indicates transfected cells, blue DAPI nuclear stain. **B**: Quantification of percentage of neurite bearing cells. A neurite was defined as being two times the cell body length. **C**: Average neurite length was also quantified. For this all extensions however small were measured. The bar graphs in B and C represent the mean values of *n* = 3, with 60 cells in each repeat, and error bars represent standard error of the mean. Statistical differences are indicated as ^**^ *P* < 0.01 or ^***^ *P* < 0.001 (one‐way ANOVA, followed by Student's *t*‐test using a Bonferroni adjusted *P* value).

Activation of the different signaling pathways triggered by TRKA ultimately results in differentiation toward a neuronal phenotype [Huang and Reichardt, 2003]. Consequently, we looked at the effect of selected mutations on induction of neuronal differentiation. The neuroblastoma cell line SH‐SY5Y was transfected with wild‐type TRKA‐GFP, the mutants p.G517E, p.L657P, p.C752S, p.C763S and empty vector and treated with NGF over 9 days (Fig. 5A).

After 9 days of NGF stimulation, 40% of cells transfected with wild‐type TRKA‐GFP differentiated into a neuronal phenotype (Fig. 5B) and the average neurite length was 360 AU (Fig. 5C). Although there was a significant reduction in the percentage of differentiated p.G517E transfected cells (^***^ *P* < 0.001), the average neurite length was not significantly different. p.C752S did not affect neurite outgrowth nor neurite length. The p.C763S mutation resulted in a significant reduction in the percentage of differentiated cells to 20% (^***^ *P* < 0.001) and a significant reduction of neurite length (^***^ *P* < 0.001). p.L657P was included as a negative control and only 5% of transfected cells could be classified as differentiated, which resembles the percentage of spontaneous differentiated cells in the empty transfected control.

### Structural Modeling of TRKA Mutations

We next modeled the structural consequences of the mutations on the kinase domain (Supp. Figs. S2 and S3C). We found that p.G517 and p.G522 are the first and third glycine residues within the ATP binding domain GxGxxG. Ligand binding and autophosphorylation of TRKA induce a conformational change that shifts TRKA into an open conformation and allows binding of ATP which increases the kinase activity [Jullien *et al*. 2002]. p.L657 is structurally within the active site of TRKA, where it is likely to have a stabilization role. p.I699 is within the activation loop, which is essential for autophosphorylation and hence may have a role in enabling autophosphorylation to occur. p.C752, p.C763, and p.R771 are all within the catalytic domain of TRKA and hence are likely to affect activity of TRKA.

## Discussion

In this study, we report seven novel missense mutations found in patients with a HSAN IV phenotype. We have undertaken a comprehensive assessment of the effects of each of these mutations on TRKA function, using a greater range of methods than has been previously reported [Greco *et al*. 1999; Greco *et al*. 2000; Mardy *et al*. 2001; Miranda *et al*. 2002]: glycosylation and membrane expression, autophosphorylation, Y496 phosphorylation, PLCγ activation and effects on neurite outgrowth (summarized in Table 1).

Glycosylation of TRKA is essential for its function and thus we assayed this for each of our mutations. p.G517E showed a similar glycosylation pattern to wild‐type TRKA‐GFP. We observed no changes in subcellular localization for this mutant protein as full glycosylation is required for trafficking to the plasma membrane [Watson *et al*. 1999]. p.G517E did not affect autophosphorylation and as a result downstream phosphorylation of Y496 was found to be unaffected. However, the p.G517E mutation completely abolished PLCγ signaling and we found that the mutation was less efficient at promoting differentiation and neurite outgrowth. p.G517E is the first glycine in the ATP binding domain GxGxxG. Hence it is surprising that Y496 phosphorylation is observed and PLCγ signaling is abolished. The attenuation of PLCγ signaling may be due a conformational change within the kinase domain that alters PLCγ signaling. This is the first mutation identified within the kinase domain that does not alter kinase activity of TRKA. It is also the first mutation identified to date that only affects one signaling pathway and yet is sufficient to restrict differentiation and thus must be the pathogenic mechanism behind HSAN IV. The result highlights the fact that when assessing the pathogenicity of TRKA patient mutations, it is not sufficient to just look at autophosphorylation or Y496 phosphorylation as has been done previously [Greco *et al*. 1999; Greco *et al*. 2000; Mardy *et al*. 2001; Miranda *et al*. 2002]. Clinically the patient with p,G517E mutation had typical HSAN IV phenotype and was indistinguishable from our other cases.

p.G522E mutation also showed a similar glycosylation pattern to wild‐type TRKA and hence no differences in membrane expression were observed. There was no induction of TRKA autophosphorylation after NGF stimulation. As a consequence of a lack of TRKA activation, downstream signaling through Y496 and PLCγ were abolished. As this mutant protein retains membrane localization, it is likely that the primary pathogenic mechanism is absent intrinsic kinase activity. The effect of this mutation is unexpected because p.G522E resides within the ATP binding domain and autophosphorylation is not dependent on ATP binding. Hence it appears that G522 may function directly in kinase activity and changes to the structure of the kinase domain leads to no kinase function. This is confirmed by studies on a different mutation at this site, p.G522R (termed p.G516R in the article) where the mutation also rendered the protein kinase‐dead [Mardy *et al*. 2001].

p.L657P, p.I699T, and p.R771C all exhibited similar results in the assays performed. We found a decrease of fully glycosylated TRKA‐GFP in these mutant proteins and the partially glycosylated 140 kDa product was favored. We hypothesized this effect may be explained by mutation‐induced conformational changes that alter the accessibility of enzymes to the glycosylation sites. An alternative explanation could be that these mutations cause aberrant folding of the TRKA kinase domain, that may be detected by cellular proofing systems and start degrading these mutants even before they become fully glycosylated. Furthermore, these mutations had more TRKA‐GFP protein in the cytoplasm than at the plasma membrane, confirming previous results that trafficking to the plasma membrane is related to glycosylation status. However, as there was some TRKA‐GFP at the membrane for each of these mutations, we next sought to investigate downstream signaling pathways. In all cases, TRKA‐GFP autophosphorylation was abolished. Neurite outgrowth in p.L657P (as a representative) was also abolished. As the four kinase‐dead mutants retained some membrane localization, it is likely that the primary pathogenic mechanism is absent intrinsic kinase activity. Structural modeling showed that p.G522E, p.L657P, p.I699T, and p.R771C were located in important parts of the kinase domain: the ATP binding domain, active site, activation loop, and the catalytic domain, respectively. It is likely that the mutations introduce a change to the 3D structure of the kinase domain which would lead to a lack of kinase activity. As such, it is unsurprising that autophosphorylation is affected and our results confirm that these residues have an essential role in autophosphorylation. These results are similar to those published previously where most the missense mutations were found to produce kinase‐dead mutants [Greco *et al*. 1999; Greco *et al*. 2000; Mardy *et al*. 2001; Miranda *et al*. 2002].

C752S did not affect glycosylation and hence did not affect trafficking to the membrane. In addition, autophosphorylation, Y496 phosphorylation and PLCγ activation were unaffected. Furthermore, neurite outgrowth data indicated that the mutation did not affect differentiation. p.C752S is within the catalytic domain but we could find no evidence of disrupted function. This heterozygous mutation was found in trans with the heterozygous kinase‐dead p.G522E mutation and no other *NTRK1* change was detected despite complete Sanger sequencing. Furthermore, the nucleotide change was not predicted to change or create a splice site. Thus, it appears that even though this mutation affects an evolutionary conserved residue, it is not pathogenic and in fact could be a rare but harmless polymorphism. In this family, we have failed to discover the other pathogenic mutation in *NTRK1*. The C752S result highlights the weakness of in silico predictions of pathogenicity and indicates functional work is necessary to call for pathogenic mutations.

p.C763S is within the catalytic domain and only partially reduced both autophosphorylation and consequently Y496 phosphorylation. Nonetheless this is sufficient to cause disease. This suggests that there is an essential level of activation of TRKA required before nociceptor differentiation can take place. As we found this patient to be phenotypically indistinguishable from our other HSAN IV cases, it must be concluded that the mutations renders the TRKA signal insufficient to trigger differentiation. This is the first reported case where a reduction in TRKA signaling, but not abolition, leads to a HSAN IV phenotype.

In conclusion, we report seven novel *NTRK1* mutations in individuals with a diagnosis of HSAN IV, and have found evidence to support pathogenicity in six. Our results highlight the fact that when assessing the pathogenicity of TRKA mutations (for example p.G517E), it is not sufficient to only assess autophosphorylation or Y496 phosphorylation, as has been done previously [Greco *et al*. 1999; Greco *et al*. 2000; Mardy *et al*. 2001; Miranda *et al*. 2002]. Similarly complete evolutionary conservation is also not an absolute predictor of pathogenicity, as we found no evidence that p.C752S was pathogenic, confirming the need for caution when interpreting the pathogenicity of unknown variants [Waxman *et al*. 2014]. Our data highlights the complexity of TRKA function and suggests that perturbation of any part of the downstream signaling may lead to a loss of nociceptor development.

*Disclosure statement*: The authors declare no conflict of interest.

## Supporting information

Disclaimer: Supplementary materials have been peer‐reviewed but not copyedited.

Supp. Fig. S1: Activity of GFP‐tagged and untagged wild‐type TRKA
Supp. Fig. S2: TrkA structure and position of mutated residues
Supp. Fig. S3: Alignment and conservation of mutated residues
Supp. Fig. S4: Colocalization of TRKA with Na+/K+ ATPase
Supp. Fig. S5: Activation of the PLCγ pathway by TRKA mutants
Supp. Table S1: Summary of functional characterization of previously identified TRKA mutations

## References

[humu23123-bib-0001] Amano A , Akiyama S , Ikeda M , Morisaki I . 1998 Oral manifestations of hereditary sensory and autonomic neuropathy type IV. Congenital insensitivity to pain with anhidrosis. Oral Surg Oral Med Oral Pathol Oral Radiol Endod 86:425–431. 9798226 10.1016/s1079-2104(98)90368-7

[humu23123-bib-0002] Artim SC , Mendrola JM , Lemmon MA . 2012 Assessing the range of kinase autoinhibition mechanisms in the insulin receptor family. Biochem J 448:213–220. 22992069 10.1042/BJ20121365 PMC3492919

[humu23123-bib-0003] Axelrod FB . 2002 Hereditary sensory and autonomic neuropathies. Familial dysautonomia and other HSANs. Clin Auton Res 12(Suppl 1):12–14. 10.1007/s102860200014 12102459

[humu23123-bib-0004] Adzhubei IA , Schmidt S , Peshkin L , Ramensky VE , Gerasimova A , Bork P , Kondrashov AS , Sunyaev SR . 2010 A method and server for predicting damaging missense mutations. Nat Methods 7:248–249. 20354512 10.1038/nmeth0410-248 PMC2855889

[humu23123-bib-0005] Bannwarth B , Kostine M . 2014 Targeting nerve growth factor (NGF) for pain management: what does the future hold for NGF antagonists? Drugs 74:619–626. 24691709 10.1007/s40265-014-0208-6

[humu23123-bib-0006] Barker PA , Lomen‐Hoerth C , Gensch EM , Meakin SO , Glass DJ , Shooter EM . 1993 Tissue‐specific alternative splicing generates two isoforms of the trkA receptor. J Biol Chem 268:15150–15157. 8325889

[humu23123-bib-0007] Bar‐On E , Weigl D , Parvari R , Katz K , Weitz R , Steinberg T . 2002 Congenital insensitivity to pain. Orthopedic manifestations. J Bone Joint Surg Br 84:252–257. 11922368 10.1302/0301-620x.84b2.11939

[humu23123-bib-0008] Beiglman A , Levy J , Hadad N , Pinsk V , Haim A , Fruchtman Y , Levy R . 2009 Abnormal neutrophil chemotactic activity in children with congenital insensitivity to pain with anhidrosis (CIPA): the role of nerve growth factors. Clin Immunol 130:365–372. 18955016 10.1016/j.clim.2008.09.005

[humu23123-bib-0009] Cunningham ME , Stephens RM , Kaplan DR , Greene LA . 1997 Autophosphorylation of activation loop tyrosine regulates signaling by the TRK nerve growth factor receptor. J Biol Chem 272:10957–10967. 9099755 10.1074/jbc.272.16.10957

[humu23123-bib-0010] Cunningham ME , Greene LA . 1998 A function‐structure model for NGF‐activated Trk. EMBO J 17:7282–7293. 9857185 10.1093/emboj/17.24.7282 PMC1171074

[humu23123-bib-0011] Datta SR , Brunet A , Greenberg ME . 1999 Cellular survival: a play in three Akts. Genes Dev 13:2905–2927. 10579998 10.1101/gad.13.22.2905

[humu23123-bib-0012] Dikic I , Batzer AG , Blaikie A , Obermeier A , Ullrich A , Schlessinger J , Margolis M . 1995 Shc binding to nerve growth factor receptor is mediated by the phosphotyrosine interaction domain. J Biol Chem 270:15125–15129. 7541035 10.1074/jbc.270.25.15125

[humu23123-bib-0013] Dubin A , Patapoutian A . 2010 Nociceptors: the sensors of the pain pathway. J Clin Invest 120:3760–3772. 21041958 10.1172/JCI42843 PMC2964977

[humu23123-bib-0014] Greco A , Villa R , Tubino B , Romano L , Penso D , Pierotti MA . 1999 A novel NTRK1 mutation associated with congenital insensitivity to pain with anhidrosis. Am J Hum Genet 64:1207–1210. 10090906 10.1086/302319 PMC1377845

[humu23123-bib-0015] Greco A , Villa R , Fusetti L , Orlandi R , Pierotti MA . 2000 The Gly571Arg mutation, associated with the autonomic and sensory disorder congenital insensitivity to pain with anhidrosis, causes the inactivation of the Ntrk1/ nerve growth factor receptor. J Cell Physiol 182:127–133. 10567924 10.1002/(SICI)1097-4652(200001)182:1<127::AID-JCP14>3.0.CO;2-0

[humu23123-bib-0016] Hepburn L , Prajsnar TK , Klapholz C , Moreno P , Loynes CA , Ogryzko NV , Brown K , Shiebler M , Hegyi K , Antrobus R , Hammond KL , Connolly J et al. 2014 Innate immunity. A Spaetzle‐like role for nerve growth factor β in vertebrate immunity to Staphylococcus aureus. Science 346:641–646. 25359976 10.1126/science.1258705 PMC4255479

[humu23123-bib-0017] Huang EJ , Reichardt LF . 2003 Trk receptors: roles in signal transduction. Annu Rev Biochem 72:609–642. 12676795 10.1146/annurev.biochem.72.121801.161629

[humu23123-bib-0018] Ismail EA , Al‐Shammari N , Anim JT , Moosa A . 1998 Congenital insensitivity to pain with anhidrosis: lack of eccrine sweat gland innervation confirmed. J Child Neurol 13:243–246. 9620018 10.1177/088307389801300511

[humu23123-bib-0019] Jullien J , Guili V , Reichardt LF , Rudkin BB . 2002 Molecular kinetics of nerve growth factor receptor trafficking and activation. J Biol Chem 277:38700–38708. 12055187 10.1074/jbc.M202348200 PMC2693056

[humu23123-bib-0020] Kumar P , Henikoff S , Ng PC . 2009 Predicting the effects of coding non‐synonymous variants on protein function using the SIFT algorithm. Nat Protoc 4:1073–81. 19561590 10.1038/nprot.2009.86

[humu23123-bib-0021] Lonze BE , Riccio A , Cohen S , Ginty DD . 2002 Apoptosis, axonal growth defects, and degeneration of peripheral neurons in mice lacking CREB. Neuron 34:371–385. 11988169 10.1016/s0896-6273(02)00686-4

[humu23123-bib-0022] McWilliam H , Li W , Uludag M , Squizzato S , Park YM , Buso N , Cowley AP , Lopez R . 2013 Analysis tool web services from the EMBL‐EBI. Nucleic Acids Res 41:597–600. 10.1093/nar/gkt376 PMC3692137 23671338

[humu23123-bib-0023] MacDonald JIS , Gryz EA , Kubi CJ , Verdi JM , Meakin SO . 2000 Direct binding of the signaling adapter protein Grb2 to the activation loop tyrosines on the nerve growth factor receptor tyrosine kinase, TRKA. J Biol Chem 275:18225–18223. 10748052 10.1074/jbc.M001862200

[humu23123-bib-0024] Mardy S , Miura Y , Endo F , Matsuda I , Indo Y . 2001 Congenital insensitivity to pain with anhidrosis (CIPA): effect of TRKA (NTRK1) missense mutations on autophosphorylation of the receptor tyrosine kinase for nerve growth factor. Hum Mol Genet 10:179–188 . 11159935 10.1093/hmg/10.3.179

[humu23123-bib-0025] Marmigere F , Ernfors P . 2007 Specification and connectivity of neuronal subtypes in the sensory lineage. Nat Rev Neurosci 8:14–127. 10.1038/nrn2057 17237804

[humu23123-bib-0026] Martin‐Zanca D , Oskam R , Mitra G , Copeland T , Barbacid M . 1989 Molecular and biochemical characterization of the human trk proto‐oncogene. Mol Cell Biol 9:24–33. 2927393 10.1128/mcb.9.1.24-33.1989 PMC362141

[humu23123-bib-0027] Miranda C , Virgilio MD , Selleri S , Zanotti G , Pagliardini S , Pierotti MA , Greco A . 2002 Novel pathogenic mechanisms of congenital insensitivity to pain with anhidrosis genetic disorder unveiled by functional analysis of neurotrophic tyrosine receptor kinase type 1/ nerve growth factor receptor mutations. J Biol Chem 277:6455–6462. 11719521 10.1074/jbc.M110016200

[humu23123-bib-0028] Mitchel DJ , Blasier KR , Jeffery ED , Ross MW , Pullikuth AK , Suo D , Park J , Smiley WR , Lo KW , Shabanowitz J , Deppmann CD , Trinidad JC et al. 2012 Trk activation of the ERK1/2 Kinase pathway stimulates intermediate chain phosphorylation and recruits cytoplasmic dynein to signaling endosomes for retrograde axonal transport. J Neurosci 32:15495–15510. 23115187 10.1523/JNEUROSCI.5599-11.2012 PMC3500848

[humu23123-bib-0029] Nahorski MS , Al‐Gazali L , Hertecant J , Owen DJ , Borner GH , Chen YC , Benn CL , Carvalho OP , Shaikh SS , Phelan A , Robinson MS , Royle SJ , Woods CG . 2015 A novel disorder reveals clathrin heavy chain‐22 is essential for human pain and touch development. Brain 138:2147–2160. 26068709 10.1093/brain/awv149 PMC4511860

[humu23123-bib-0030] Obermeier A , Halfter H , Wiesmuller KH , Jung G , Schlessinger J , Ullrich A . 1993 Tyrosine 785 is a major determinant of Trk‐substrate interaction. EMBO J 12:933–941. 8384556 10.1002/j.1460-2075.1993.tb05734.x PMC413294

[humu23123-bib-0031] Riccio A , Ahn S , Davenport CM , Blendy JA , Ginty DD . 1999 Mediation by a CREB family transcription factor of NGF‐dependent survival of sympathetic neurons. Science 286:2358–2361. 10600750 10.1126/science.286.5448.2358

[humu23123-bib-0032] Rosemberg S , Marie SK , Kliemann S . 1994 Congenital insensitivity to pain with anhidrosis (hereditary sensory and autonomic neuropathy type IV). Pediat Neurol 11:50–56. 7527213 10.1016/0887-8994(94)90091-4

[humu23123-bib-0033] Sali A , Blundell TL . 1993 Comparative protein modelling by satisfaction of spatial restraints. J Mol Biol 234:779–815. 8254673 10.1006/jmbi.1993.1626

[humu23123-bib-0034] Spierings EL , Fidelholtz J , Wolfram G , Smith MD , Brown MT , West CR . 2014 A phase III placebo‐ and oxycodone‐controlled study of tanezumanb in adults with osteoarthritis pain of the hip or knee: response. Pain 155:2432–2333. 10.1016/j.pain.2014.08.038 25194672

[humu23123-bib-0035] Veale EL , Al‐Moubarak E , Bajaria N , Omoto K , Cao L , Tucker SJ , Stevens EB , Mathie A . 2014 Influence of the N terminus on the biophysical properties and pharmacology of TREK1 potassium channels. Mol Pharmacol 85:671–81. 24509840 10.1124/mol.113.091199

[humu23123-bib-0036] Watson FL , Porcionatto AM , Bhattacharyya A , Stiles CD , Segal RA . 1999 TRKA glycosylation regulates receptor localization and activity. J Neurobiol 39:323–36. 10235685 10.1002/(sici)1097-4695(199905)39:2<323::aid-neu15>3.0.co;2-4

[humu23123-bib-0037] Waxman SG , Merkies IS , Gerrits MM , Dib‐Hajj SD , Lauria G , Cox JJ , Wood JN , Woods CG , Drenth JP , Faber CG . 2014 Sodium channel genes in pain‐related disorders: phenotype‐genotype associations and recommendations for clinical use. Lancet Neurol 13:1152–1160. 25316021 10.1016/S1474-4422(14)70150-4

[humu23123-bib-0038] Yuang J , Yankner BA . 2000 Apoptosis in the nervous system. Nature 407:802–809. 11048732 10.1038/35037739

