# Supplementary material for: A Comprehensive Functional Analysis of NTRK1 Missense Mutations Causing Hereditary Sensory and Autonomic Neuropathy Type IV (HSAN IV)
Source: Hum Mutat. 2016 Nov 26;38(1):55–63. doi: 10.1002/humu.23123 (PMC5299464; doi:10.1002/humu.23123)
Supplement: Supplementary file 1 — Supp. Fig. S1: Activity of GFP‐tagged and untagged wild‐type TRKA Supp. Fig. S2: TrkA structure and position of mutated residues Supp. Fig. S3: Alignment and conservation of mutated residues Supp. Fig. S4: Colocalization of TRKA with Na+/K+ ATPase Supp. Fig. S5: Activation of the PLCγ pathway by TRKA mutants Supp. Table S1: Summary of functional characterization of previously identified TRKA mutations [file HUMU-38-55-s001.pdf]

Supplementary Figure S1: Activity of GFP-tagged and untagged wild-type TRKA

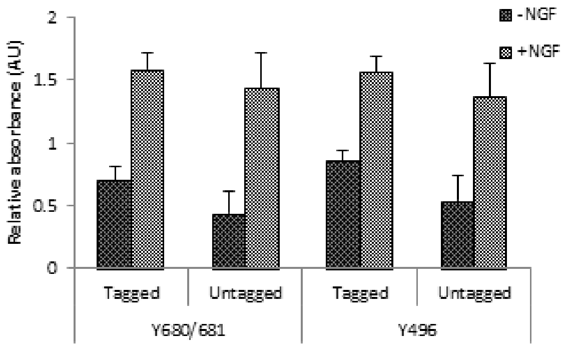

To test whether the introduction of GFP at the C-terminus of TRKA affected its function, we introduced the stop codon, TAG, into the construct using site directed mutagenesis and introduction of the mutation was confirmed by sequencing. HeLa cells were then transfected with the two different constructs, and were treated with NGF or were left untreated. Y680/681 autophosphorylation and Y496 phosphorylation were determined by ELISA. There was no observable differences in the basal levels of autophosphorylation or Y496 phosphorylation in cells transfected with GFP-tagged and untagged constructs (P= 0.40, P= 0.37, respectively, n= 3, Student's t test). Furthermore, NGF-induced autophosphorylation and Y496 phosphorylation were not different in cells transfected with GFP-tagged and untagged constructs (P= 0.85, P= 0.90, respectively, n= 3, Student's t test).

Supplementary Figure S2: TrkA structure and position of mutated residues

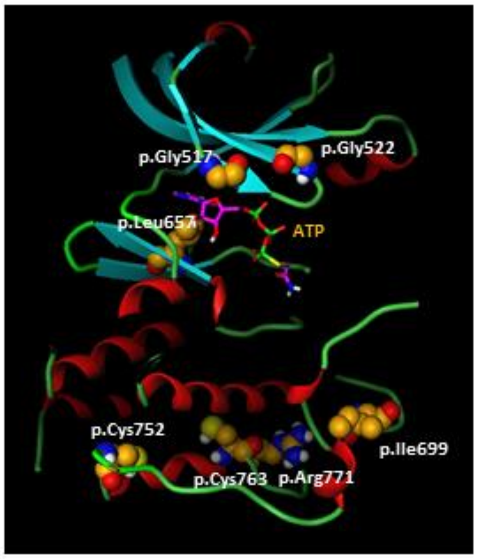

According to the structural model of the kinase domain of TRKA, p.G517, p.G522 and p.L657, located in the ATP binding domain and the active site, are in close proximity to ATP. Any alteration to these residues is likely to interfere with interaction with ATP and may lead to structural alterations. p.I699 is located within the activation loop and the three remaining residues are within the catalytic domain and possibly are required for substrate binding.

Supplementary Figure S3: Alignment and conservation of mutated residues

A

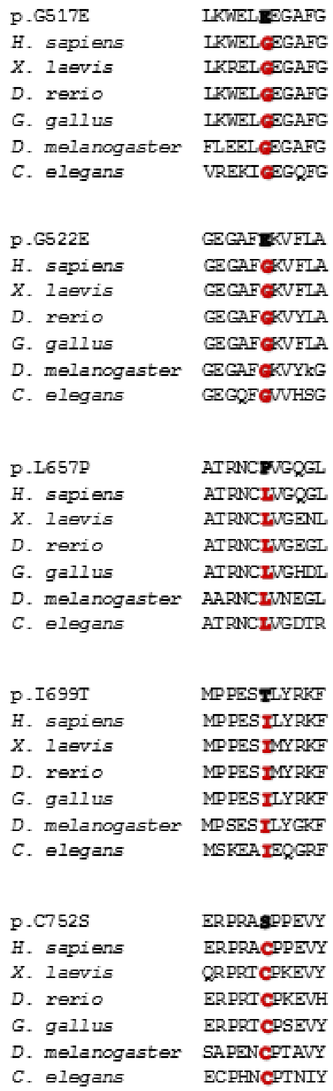

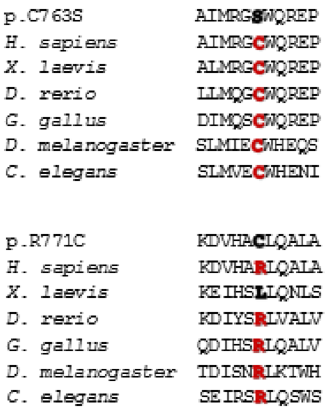

B

Mutation	SIFT prediction score	Polyphen prediction score
p.G517E	0.00	1.000
p.G522E	0.00	1.000
p.L567P	0.00	0.983
p.I699T	0.00	0.953
p.C752S	0.00	1.000
p.C763S	0.00	1.000
p.R771C	0.00	1.000

C

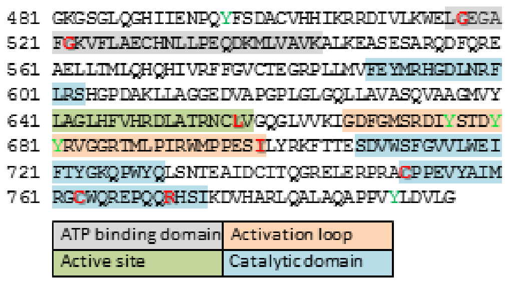

(A): The missense TRKA candidate mutations all resulted in the change of an evolutionary conserved amino acid. Protein sequence data are for human as a representative mammal (TKRA, NP_00252), *Xenopus laevis* for amphibians (NP 001079579.1), *Danio rerio* for fish (NP_001288285), *Drosophila melanogaster* for flies (NP_476962.1) and *Caenorhabditis elegans* as a nematode (NP_001033327.1). Multiple sequence alignment was performed using Clustl Omega. The candidate mutations are shown in black and the amino acid at that

position in the different species are shown in red. Any variance that occurs at this position is also shown in black.

(B): All of the missense mutations were predicted to be damaging and pathogenic by Polyphen, where most damaging is given a score of 1. These same mutations were also predicted to be damaging by SIFT with the lowest score of 0.

(C): The identified residues are all within in subdomains of the tyrosine kinase domain of TRKA as shown.

Supplementary Figure S4: Colocalization of TRKA with Na^+/K^+ ATPase

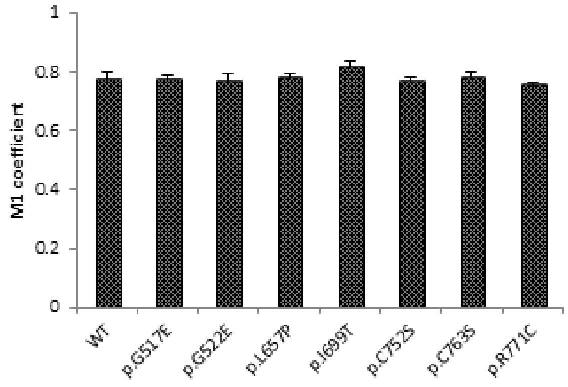

Colocalization of TRKA at the periphery of the cells with the plasma membrane Na^+/K^+ ATPase was determined in terms of the Manders M1 coefficient. Wild-type TRKA was found to colocalize with the plasma membrane as were all the mutant TRKA proteins. WT: wild-type.

Supplementary Figure S5: Activation of the PLCγ pathway by TRKA mutants

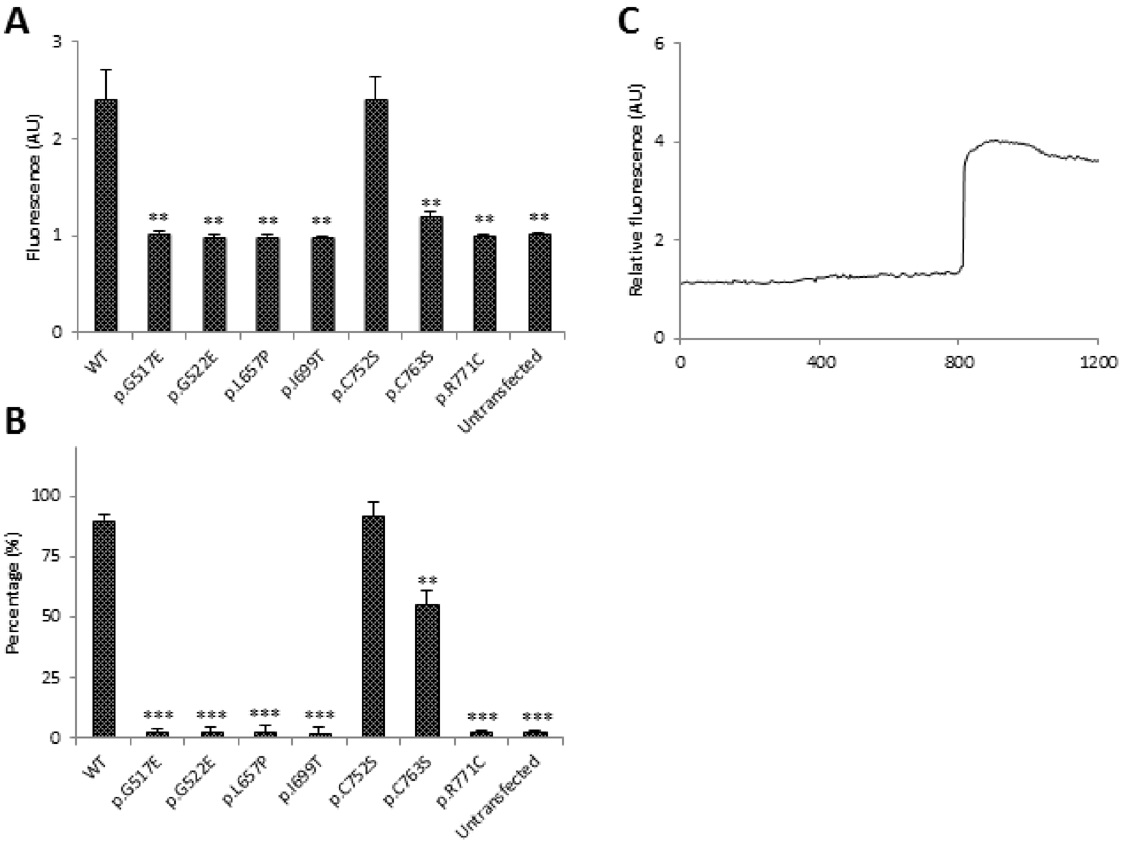

(A): HeLa cells were transfected and the calcium levels were monitored after NGF application. Fluorescence at t= 208 s was quantified. The maximum fluorescence in C752S transfected cells was not different from the maximum fluorescence of wild-type transfected cells. Maximum fluorescence was reduced 2-fold in C763S transfected cells and in all the other mutants it did not increase above baseline levels. WT: wild-type.

(B): The percentage of cells with a calcium response was determined. Any cell that had a relative fluorescence greater than 15% of the baseline after NGF addition was classified as a responsive cell. A 90% of wild-type and C752S cells had a calcium response, whereas this was reduced in C763s transfected cells and was almost 0% in all the other mutants.

The bar graphs in A and B represent the mean values of n= 3, with around 15 cells in each repeat, and error bars represent standard error of the mean. Statistical differences between wild-type and mutant TRKA are indicated as **$P<0.01$ or ***$P<0.001$ (one-way ANOVA, followed by Student's t test using a Bonferroni adjusted P-value).

(C): WT transfected HeLa cells were treated with 3 μM of U73122, a phospholipase C inhibitor for 30 min. After incubation, the NGF induced increase in fluorescence was inhibited.

Supplementary Table S1: Summary of functional characterization of previously identified TRKA mutations

Mutation	Full glycosylation	Membrane expression	Autophos-phorylation	Y496 phos-phorylation	PLCγ activation	Neurite outgrowth
p.L93P	Abolished	—	Abolished	Abolished	—	—
p.L213P	Abolished	Null	Abolished	Abolished	—	Abolished
p.G516R	No effect		Abolished	Abolished	—	—
p.G571R	—	No effect	Abolished	Abolished	—	Abolished
p.R643W	No effect	—	Abolished	Abolished	—	Abolished
p.R648C	No effect	—	Abolished	Abolished	—	—
p.G708S	No effect	—	Abolished	Abolished	—	Abolished
p.R774P	—	—	Abolished	—	—	—

Functional characterization of previously published mutations identified these as functional nulls, where kinase activity was abolished. In most cases Y496 phosphorylation was also investigated as was the presence of the fully glycosylated form.

Supplementary Table S2: Summary of phenotype of patients

Case	*NTRK1* mutations	TRKA protein	Age at diagnosis	Ethnicity and sex	Congeital analgaesia	Sweating	Temperature sensing/ episodes of hyperthermia	Itching/ and licheni-fication	Cognition	Staphylococcal Aureus infections/ Charcot's joints
1	Het c.1550G>A, c.717+4A>T[a]	p.G517E, Null	15y	Caucasian, male	Yes	None	None/No	No/No	Mild cognitive delay	Yes/yes
2	Het c.1565G>A, c.2254T>A	p.G522E, p.C752S	8y	Caucasian, female	Yes	None	None/No	Yes/No	Mild cognitive delay	Yes/ No
3 and 4	Homozygous c.1970T>C	p.L657P	10y and 3y	Pakistani*, female and male	Yes	None	None/No	Yes/Yes and Yes/No	Moderate cognitive delay and ?	Yes/Yes and Yes/No
5	Het c.2096T>C, c.287+2 dupT[b]	p.I699T, Null	3y	Pakistani, male	Yes	None	None/Yes	Yes/No	Mild cognitive delay	Yes/No
6	Homozygous c.2288G>C	p.C763S	6y	Turkish*, Male	Yes	None	None/Yes	Yes/No	Mild cognitive delay	Yes/No
7	Homozygous c.2311C>T	R771C	8y	Indian*, male	Yes	None	None/Yes	Yes/Yes	Moderate cognitive delay	Yes/No

Nucleotide numbering uses +1 as the A of the ATG translation imitation codon in the reference sequence, with the initiation codon as codon 1 (RefSeq ID NM_002529.3, NP_002520) .

a. Alters an evolutionary conserved splice donor site of intron 6 from gGTAAT to gGTATT, where the +4 A is conserved in all species with a NTRK1 gene. Of note the spice acceptor for this intron 5 of NTRK1 is predicted as poor TAACACCCCTTGGCCCTCGGCGTCCTGGGTGGCCAGg. MIT splice predictor, SplicePort and NetGene2 – do not predict the proven NTRK1 intron 5 acceptor splice site.

b. Changing the splice donor site from tGTGAG to tGTTGAG; previously reported as pathogenic in Lee ST et al, Muscle Nerve, 2009, 40(5):855-9)

*. Parents consanguineous.
